# Combined Therapy Targeting MET and Pro-HGF Activation Shows Significant Therapeutic Effect Against Liver Metastasis of CRPC

**DOI:** 10.3390/ijms26052308

**Published:** 2025-03-05

**Authors:** Shoichi Kimura, Satoshi Iwano, Takahiro Akioka, Takahiro Kuchimaru, Makiko Kawaguchi, Tsuyoshi Fukushima, Yuichiro Sato, Hiroaki Kataoka, Toshiyuki Kamoto, Shoichiro Mukai, Atsuro Sawada

**Affiliations:** 1 Department of Urology, Faculty of Medicine, University of Miyazaki, Miyazaki 889-1692, Japan; 2 Institute for Tenure Track Promotion, University of Miyazaki, Miyazaki 889-1692, Japan; 3 Center for Molecular Medicine, Jichi Medical University, Tochigi 329-0498, Japan; 4 Section of Oncopathology and Morphological Pathology, Department of Pathology, Faculty of Medicine, University of Miyazaki, Miyazaki 889-1692, Japan; kawaguchi@med.miyazaki-u.ac.jp (M.K.);; 5 Organization for Promotion of Research and Industry Academic Regional Collaboration, University of Miyazaki, Miyazaki 889-1692, Japan

**Keywords:** CRPC, liver metastasis, HGF, MET

## Abstract

The liver is the most lethal metastatic site in castration-resistant prostate cancer (CRPC). Overexpression of MET protein has been reported in CRPC, and *MET* is an important driver gene in androgen-independent CRPC cells. Mouse CRPC cell line CRTC2 was established by subcutaneous injection of hormone-sensitive PC cells (TRAMP-C2) in castrated nude mice. CRCT2/luc2 cells were injected into the spleen of castrated nude mice, and liver metastasis was confirmed at 2 weeks post-injection. We administered MET inhibitor (MET-I) and HGF activator inhibitor (HGFA-I) to this liver metastasis model and assessed the therapeutic effect. After intrasplenic injection, CRTC2 showed a higher incidence of liver metastasis whereas no metastasis was observed in TRAMP-C2. Microarray analysis revealed increased expression of *HGF*, *MET*, and *HPN*, *HGFAC* (encoding HGF activating proteases) in liver metastasis. Proliferation of CRCT2 was significantly inhibited by co-administration of MET-I and HGFA-I by in vitro analysis with HGF-enriched condition. In an analysis of the mouse model, the combination-therapy group showed the strongest reduction for liver metastasis. Immunohistochemical staining also revealed the strongest decrease in phosphorylation of MET in the combination-therapy group. Co-culture with HGF-expressed mouse fibroblasts showed attenuation of the inhibitory effect of MET-I; however, additional HGFA-I overcame the resistance. We established an androgen-independent CRPC cell line, CRTC2, and liver metastasis model in mice. Significant effect was confirmed by combined treatment of MET-I and HGFA-I by in vitro and in vivo analysis. The results suggested the importance of combined treatment with both MET- and HGF-targeting agents in the treatment of HGF-enriched conditions including liver metastasis.

## 1. Introduction

Prostate cancer (PC) is the most common cancer in males, accounting for 15% of all male cancers [1]. Approximately 10% of PC patients have distant metastasis at the initial diagnosis [2,3,4]. Common metastatic sites are bone and lymph nodes. Although visceral metastasis of PC has been reported as a poor prognostic factor for overall survival (OS), metastasis is uncommon in hormone sensitive PC (HSPC) [2,5,6]. Visceral metastasis may occur during or after treatment with androgen deprivation therapy (ADT), androgen receptor signaling inhibitors (ARSi), and chemotherapy, and it commonly appears as castration resistant PC (CRPC) [2,5,6]. A meta-analysis of phase III trial patients with CRPC receiving docetaxel revealed a rate of 20.8% visceral metastasis [2]. Among patients with metastatic CRPC, liver metastasis was observed in 8.6%, and it had the worst median OS at 13.5 months compared with bone (21.3 months) and lung metastasis (19.4 months) [5]. An analysis of biomarkers, including circulating DNA, in patients with CRPC demonstrated that patients with a high concentration of serum carcinoembryonic antigen (CEA) had higher rates of liver metastasis, and that patients showed a higher likelihood of harboring copy number amplification in *MET*, a specific receptor of hepatocyte growth factor (HGF) [7].

In the pericancerous microenvironment, HGF is mainly secreted from stromal cells, including cancer-associated fibroblasts (CAF), as an inactive pro-form (pro-HGF) and proteolytically activated by the specific serine proteases, including HGF activator (HGFA, serum activator), hepsin and matriptase (the latter two are transmembrane proteases) [8,9,10]. HGF-induced activation of MET signaling axis has been reported to be involved in the progression of various cancers by upregulation of proliferation, invasive activity, motility, and anti-apoptotic activity [8,9,10]. Therefore, MET-targeting tyrosine kinase inhibitors (MET-TKI) have been used in the treatment of various cancers, including non-small cell lung cancer (NSCLC), metastatic medullary thyroid cancer, renal cell carcinoma, and hepatocellular carcinoma, with acceptable therapeutic outcomes; multiple advanced clinical trials are currently underway [11,12]. However, acquired resistance remained a serious issue [11,13,14,15]. Of interest, the pharmacological effect of MET-TKI for MET-amplified NSCLC and gastric cancer cells was predominantly reduced under HGF-enriched conditions; however, additional inhibition of active HGF overcame resistance [15]. In the study, the authors reported transformation of the cancer cells to facilitate survival, from HGF-independent to HGF-dependent type, under HGF-rich conditions. The result suggested the significance of HGF activation-targeted therapy in overcoming the resistance of the MET inhibitor.

HGF is well known as a potent mitogen for hepatocytes and is enriched in hepatic stellate cells (HSC) 16–18. In addition, the majority of HGFA and hepsin is produced in the liver [8,9,10], suggesting that the liver provides a specific microenvironment for active HGF-enriched conditions. Indeed, HGF can protect from liver injury, and the protective role of HGF occurs in healthy livers as well as in the early stage of liver disease [16,17,18]. On the other hand, the HGF/MET axis promotes the invasive growth of cancers; therefore, evaluation of HGF/MET targeted therapy is performed for hepatocellular carcinoma and cholangiocarcinoma (CCA) [17,18,19]. In the analysis of CCA and CAF, it has been reported that the majority of CAF was derived from HSC, and inflammatory CAF (iCAF)-produced HGF promoted the growth of intrahepatic CCA through activation of MET expressed in the tumor [19]. Therefore, we hypothesized that the HGF/MET axis may be a key signaling pathway in liver metastasis of MET-expressed CRPC cells, and that therapy targeting the molecules may have potential therapeutic benefits. In addition, reduction in active HGF may also be important in preventing and/or overcoming resistance to MET-TKI. In this study, we attempted to inhibit the growth of CRPC cells in liver metastasis by dual inhibition of MET phosphorylation and pro-HGF activation using a mouse model of liver metastasis.

## 2. Results

### 2.1. Establishment of Mouse CRPC Cell Line (CRTC2) and Mouse Liver Metastasis Model

**Figure 1:**
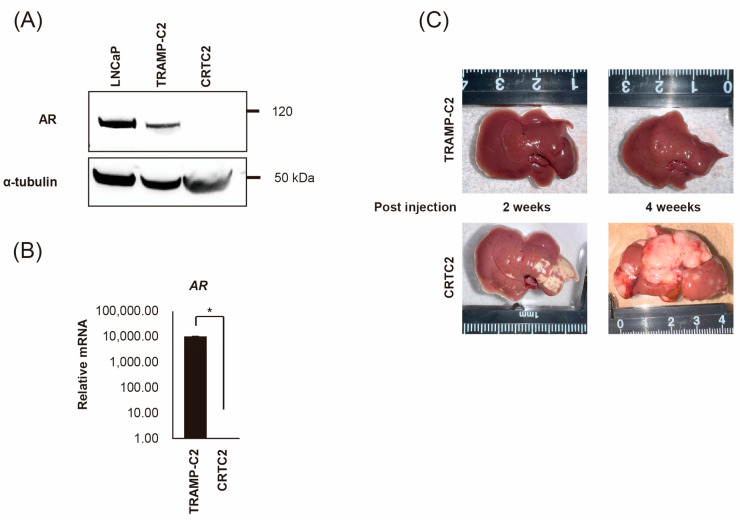
Establishment of mouse CRPC cell line (CRTC2) and the liver metastasis model. TRAMP-C2 cells were injected into the subcutaneous tissue of a female nude mouse. The growing tumor was transferred to a culture dish and cultured for five generations in the absence of androgen, then CRCT2 cells were established. (**A**) Expression of AR protein was determined for CRCT2 compared with LNCaP and TRAMP-C2 (anti-AR antibody and anti-α-tubulin antibody can react to both human and mouse protein). (**B**) Downregulation of *AR* in CRCT2 was also confirmed by real-time quantitative PCR (* *p* < 0.01). (**C**) TRAMP-C2 and CRTC2 cells (2 × 10^6^ cells/100 µL in PBS) were injected into the mouse spleen. After 2 and 4 weeks, mice were scarified. Representative macroscopic appearance of the liver is shown.

TRAMP-C2 cells were injected into the subcutaneous tissue of a female nude mouse. After 8 weeks, the tumor was transferred to a culture dish and cultured for five generations in the absence of androgen; and the mouse androgen independent CRPC cell line, CRTC2, was established [20]. First, we confirmed the expression of *AR* between TRAMP-C2 and CRTC2 cells by RT-qPCR and immunoblot analysis. As a result, expression of AR was downregulated in CRTC2 compared with TRAMP-C2 in both mRNA and protein (Figure 1A,B, degree of *AR* mRNA expression was extremely low). Next, both cells (2.0 × 10^6^/100 µL in PBS) were injected into a mouse spleen. After 2 and 4 weeks, mice were scarified, and the formation of liver metastasis was confirmed (Figure 1C) [21]. Apparent liver metastasis was confirmed in the CRTC2 group (6/6), whereas no metastasis was observed in the TRAMP-C2 group (0/6, Figure 1C). No apparent additional metastasis (in other organs, including bone) was observed in either group.

### 2.2. Comparison of mRNA Expression Between Non-Metastatic and Metastatic CRTC2

**Figure 2:**
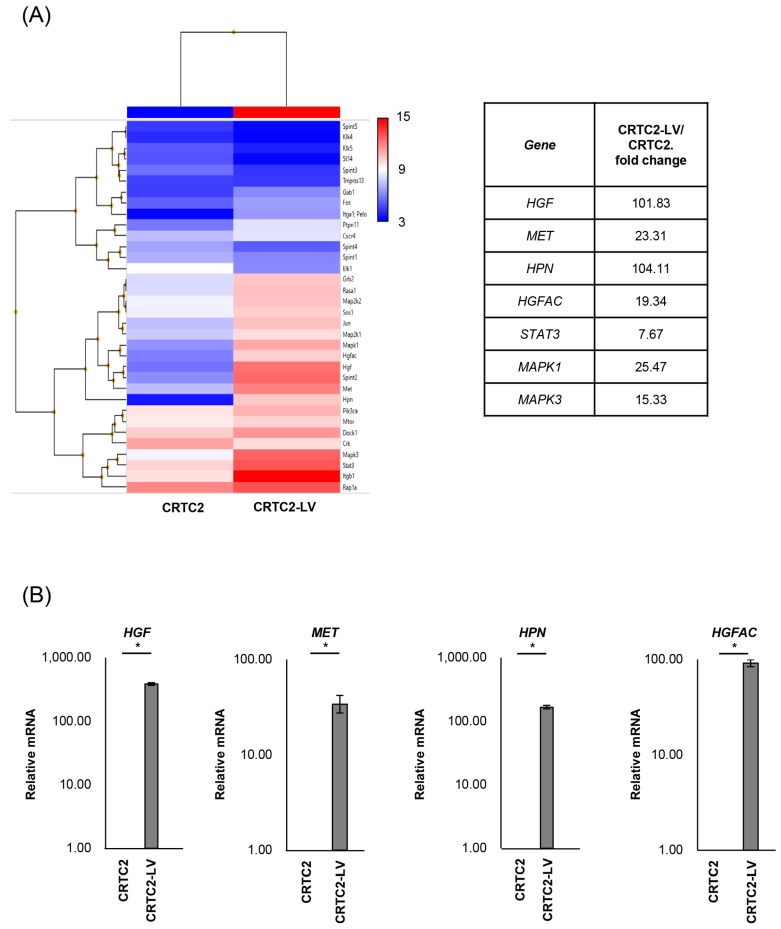
Comparison of mRNA expression between non-metastatic and metastatic CRTC2. (**A**) Results of microarray analysis of CRTC2 and the liver metastasis are shown. Microarray analysis was outsourced to Cosmo Bio Co., Ltd. (Tokyo, Japan). The company performed cDNA synthesis, labeling, hybridization, washing, and scanning processes using Clariom S Array, mouse according to their proprietary protocols and equipment. The raw data provided were normalized and analyzed using Transcriptome Analysis Console Software. (**B**) Expression of *HGF*, *MET*, *HPN* and *HGFAC* was also confirmed by real time RT-qPCR (* *p* < 0.05). The figure shows ratios to CRTC2.

The mRNA expression of cancer-related molecules, including those within the HGF/MET signaling pathway, were determined by microarray analysis, and compared between non-metastatic CRTC2 and metastatic CRTC2 (liver metastasis: CRTC2-LV) (Figure 2A). Of interest, significant upregulation of molecules related to the HGF/MET pathway, including *HGF*, *MET* (fold change of 101.83, 23.31), *HPN*, and *HGFAC* (encoding HGF activating proteases, hepsin, and HGF activator, fold change of 104.11, 19.3) in CRCT2-LV compared with non-metastatic CRCT2 (Figure 2A, right). Increased expression of *HGF*, *MET*, *HPN*, and *HGFA* was also confirmed by real time RT-qPCR (Figure 2B).

### 2.3. Inhibition of MET and HGF-Activation in CRTC2 Cells, In Vitro Analysis

**Figure 3:**
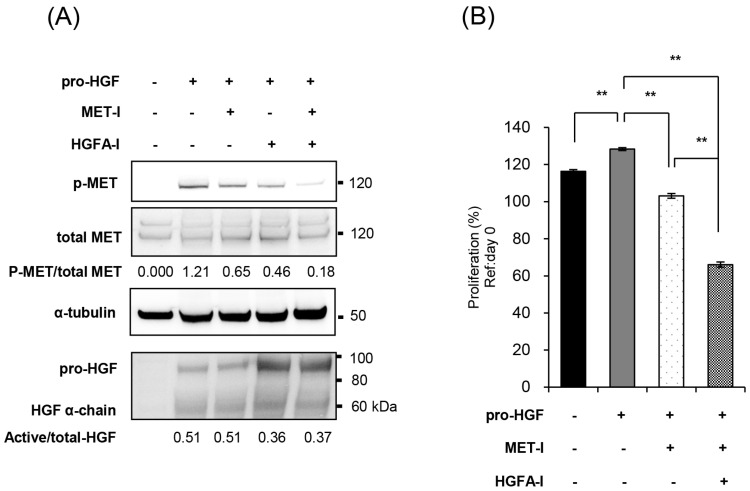
Inhibition of MET and HGF-activation in CRTC2 cells, in vitro analysis. (**A**) CRTC2 cells were pre-cultured in FBS-free DMEM for 24 h. The cells were then treated with each agent (final concentration of 250 nM for MET-I and 10 µM for HGFA-I) at 37 °C for 2 h, followed by the addition of mouse recombinant pro-HGF (50 ng/mL). After incubation at 37 °C for 2 h, the proteins were extracted. Phosphorylation of MET and expression of total-MET, HGF, and α-tubulin were determined by immunoblot analysis. (**B**) CRTC2 cells were pre-cultured in FBS-free DMEM for 24 h. The cells were seeded in a 96-well plate at a density of 1 × 10^4^ cells/100 µL in FBS-free DMEM. The cells were then treated with each agent (final concentration of 20 µM for MET-I and 20 µM for HGFA-I) at 37 °C for 2 h, followed by the addition of 50 ng/mL mouse recombinant pro-HGF, and incubated at 37 °C for 48 h. Cell proliferation was analyzed by detecting the absorbance at 490 nm and calculated as a ratio to the absorbance at day 0 (before administration, ** *p* < 0.01).

Based on these findings, we investigated the efficacy of targeting the HGF/MET pathway for the treatment of CRTC2 liver metastases. We selected JNJ-38877605 (MET-I), a highly selective MET tyrosine kinase inhibitor, and SRI31215TFA (HGFA-I), a synthetic inhibitor of pro-HGF activating proteases, and evaluated their efficacy in vitro in the presence of pro-HGF. As a result, the single use of MET-I slightly inhibited MET phosphorylation without effect on pro-HGF activation. HGFA-I inhibited activation of pro-HGF and weakly inhibited phosphorylation of MET. However, the strongest inhibition of MET phosphorylation was observed by combination of MET-I and HGFA-I (Figure 3A). Next, the effect on cell proliferation was analyzed. CRTC2 cells showed a slight increase in proliferation in the presence of pro-HGF. Although an inhibitory effect was observed by the single use of MET-I, the strongest therapeutic effect was confirmed by combination (MET-I and HGFA-I, Figure 3B).

### 2.4. Effect of Inhibition of MET and HGF-Activation in Liver Metastasis In Vivo

**Figure 4:**
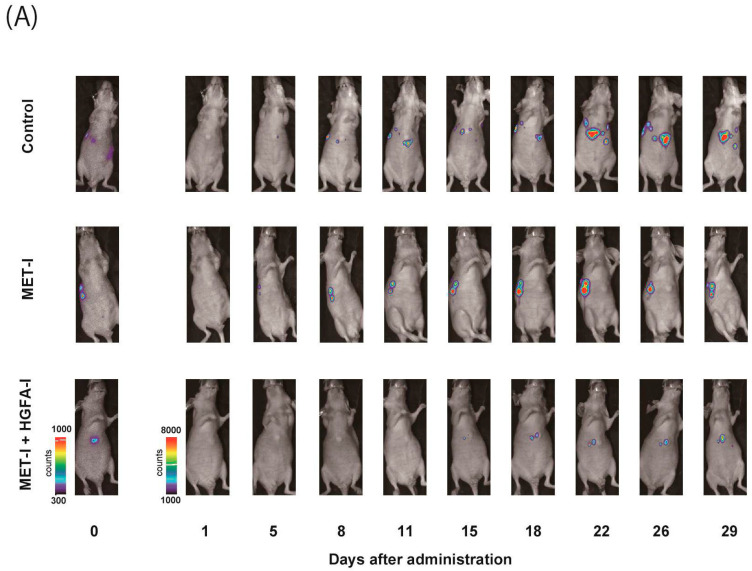

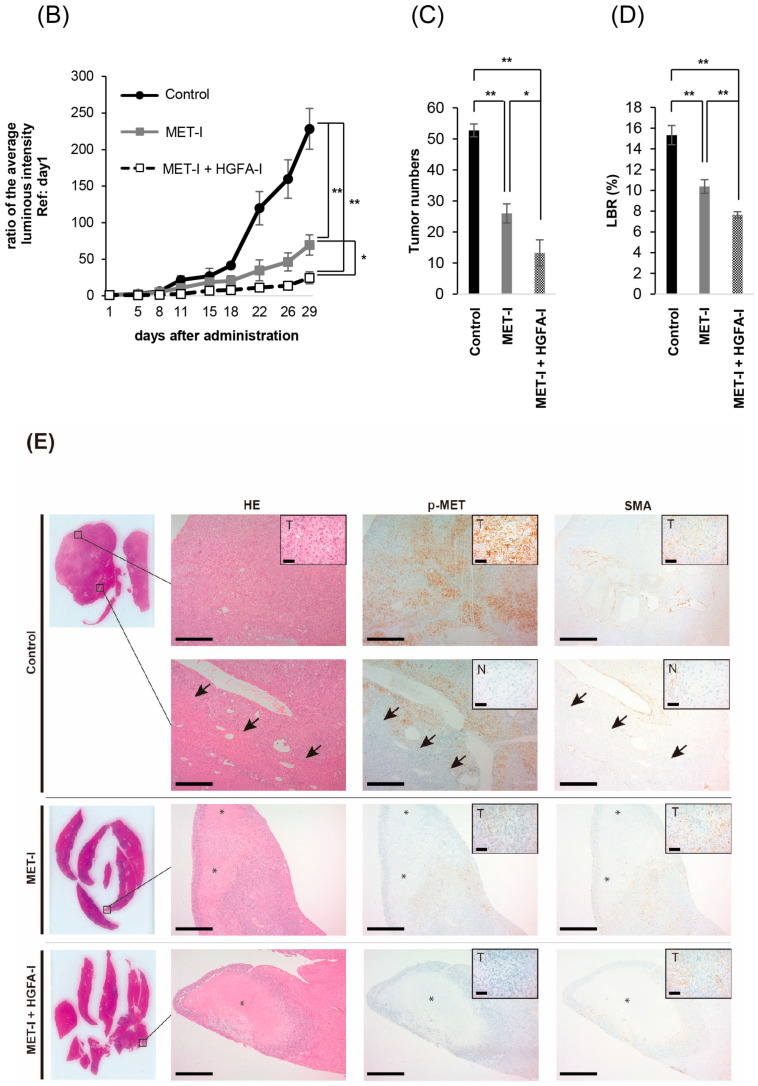
The effect of inhibition of MET and HGF-activation in liver metastasis, in vivo analysis. (**A**) A suspension of 2 × 10^6^ CRTC2-luc2 cells/100 µL in PBS was injected into the spleen of orchiectomized male nude mice. Bioimaging was used to confirm the formation of liver metastases by an injection of 100 μL of 100 mM D-luciferin. Mice with confirmed liver metastases were divided into three groups: (1) control group (vehicle), (2) a group with single use of MET-I (30 mg/kg, oral administration, every other day), and (3) a group with a combination of MET-I and HGFA-I (5 mg/kg, intravenous injection, every other day). The figure presents representative images of liver metastasis progression captured by the bioimaging device during the treatment period. (**B**) Luminescence counts were detected as the average value within the range encompassing the liver, and the temporal changes in each group are shown relative to the value on the day treatment started. Values are presented as mean ± SE (N = 4, each group). The significance was also determined by the number of metastases (**C**) and liver/body weight ratio (LBR, **D**). * *p* < 0.05, ** *p* < 0.01. (**E**) Representative histological findings are shown. Th upper two horizonal lines are control, the middle line shows single-use therapy (MET-I), and the lower line is combination-therapy (MET-I and HGFA-I). Vertical columns are, from left to right, loupe image, hematoxylin-eosin (HE) staining, immunohistochemical staining of phosphorylation of MET (p-MET) and that of smooth muscle actin (SMA). The border between cancer nests and non-malignant liver tissue (inset: N) is also shown (black arrows). Scale bar = 500 μm, 50 μm (inset). T: tumor, N: non-malignant live tissue, * necrotic area.

To evaluate the therapeutic effects of MET-I and HGFA-I on liver metastasis, we analyzed the therapeutic effect of both agents on a mouse model with liver metastasis. Luciferase-expressing CRTC2-mCherry-luc2 (CRTC2-luc2) cells were injected into the spleen of castrated male nude mice. Bioimaging was used to confirm the formation of liver metastases by injection of 100 μL of 100 mM D-luciferin. Mice with confirmed liver metastases were divided into three groups: (1) control group (vehicle), (2) group with single use of MET-I, and (3) group with combination of MET-I and HGFA-I. As shown in Figure 4A, both the single-use and combination-therapy groups showed inhibition of liver metastasis progression compared to the vehicle group. Similar to the result of in vitro analysis, the combination-therapy group showed the strongest reduction for liver metastasis compared with the single-use group. Significance was determined by difference in luminous intensity, number of metastases and liver/body weight ratio (LBR) (Figure 4B–D). The degree of luminous intensity differed in a time-dependent manner. Significant therapeutic effect was observed in both treatment groups (single and combined) compared with control, and the strongest effect was confirmed statistically in the combination-therapy group in all evaluations. The luminescence count of liver metastasis was reduced to 30.4% in the monotherapy (MET-I) group and 10.8% in the combination-therapy group compared with control group (*p* = 0.011, 0.047, respectively). Pathological evaluation was also performed. As shown in Figure 4E, large cancer nests composed of viable cancer cells were observed in the control group (upper two horizonal line, inset: T). The border between cancer nests and non-malignant liver tissue (inset: N) is also shown (black arrows). Increased phosphorylation of MET was observed in cancer cells (inset: T), whereas MET phosphorylation was downregulated in non-malignant liver tissue (inset: N). Smooth muscle actin (SMA)-positive stromal cells were diffusely presented in the peri-cancerous area; however, no apparent SMA-positive cells were observed in non-malignant liver tissue except for vessel wall (inset: N). The number and size of cancer nests were apparently reduced, and large central necrosis (*) was evident in the cancer nests of both treatment groups (column of HE, middle, and lower lines). Immunohistochemical staining revealed decreased phosphorylation of MET in treatment group compared with control (column of p-MET, middle, and lower lines). Phosphorylation of MET remained, but weakly in the single-use therapy group; however, almost complete inhibition was observed in the combination-therapy group (inset: T). SMA-positive stromal cells also remained around surviving viable cancer cells in both treatment groups (inset: T). SMA has been reported to be a marker of CAF [22]. Considered with the appearance of MET phosphorylation, it is suggested that providing HGF forms CAF and the activating system remains active in the group with the single-use MET-I; however, the system is almost completely downregulated in the group with MET-I-HGFA-I combined-therapy group.

### 2.5. Inhibition of Fibroblast-Derived HGF: Analysis by Co-Culture with Mouse Fibroblast Cell, NIH3T3

**Figure 5:**
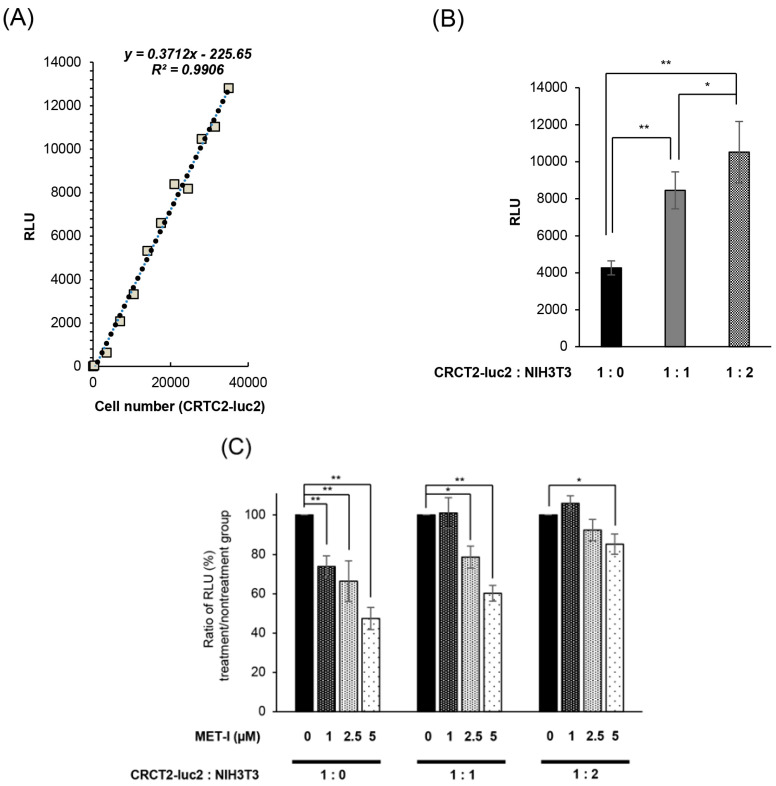

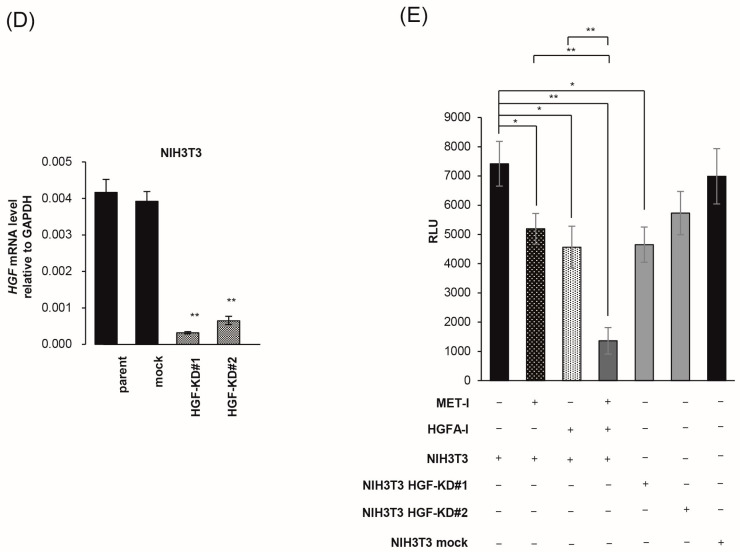
Inhibition of fibroblast-derived HGF: analysis by co-culture with mouse fibroblast cell, NIH3T3. (**A**) Reference for measurement. Luminescence was measured at 30 s after administration of D-luciferin, and the proliferation of CRCT2-luc2 was measured as the average intensity. Validation between counted cell numbers and degree of luminescence was determined (*R*^2^ values ≥ 0.990). (**B**) Proliferation of CRCT2 cells with or without NIH3T3 cells (mouse fibroblast) by degree of luminescence. Ratios of initial cell numbers of CRCT2 and NIH3T3 are shown as 1:0, 1:1, 1:2. * *p* < 0.05, ** *p* <0.01. (**C**) Therapeutic effect of MET-I with various concentration in co-culture with different degree of NIH3T3. The effect was determined by luminescence. Luminal intensity of treatment group was compared with the non-treatment group. Ratios of initial cell numbers of CRCT2 and NIH3T3 are shown as 1:0, 1:1, 1:2. * *p* < 0.05, ** *p* < 0.01. (**D**) Quantification of HGF mRNA level in NIH3T3. The result of real time quantitative PCR in each cell line is shown. ** *p* < 0.01. KD: knock down (**E**) Inhibition of CRCT2 cell proliferation by MET-I and HGFA-I. CRCT2 cells are cultured with NIH3T3, NIH3T3 HGFKD#1, NIH3T3 HGFKD#2 or NIH3T3 mock (ratio of initial cell numbers of CRCT2 and NIH3T3 is 1:2). CRTC2 cells were pre-cultured in FBS-free DMEM for 24 h. The cells were seeded in a 96-well plate at a density of 2 × 10^4^ cells/100 µL in FBS-free DMEM. The cells were then treated with each agent (final concentration of 2.5 µM for MET-I and 40 µM for HGFA-I) at 37 °C for 2 h, and incubated at 37 °C for 96 h. Cell proliferation was analyzed by detecting the absorbance at 490 nm and calculated as a ratio to the absorbance at day 0. Therapeutic effect was determined by the luminescence and statistically compared with control (co-culture with NIH3T3 without treatment). * *p* < 0.05, ** *p* < 0.01.

Next, we determined the effect of stromal cells on the growth of CRTC2 cells by co-culture with mouse fibroblast cell line NIH3T3, which expressed HGF. For accurate evaluation of cell proliferation in co-culture, we used luciferase-expressing CRTC2 (CRTC2-luc2) cells, and proliferation was analyzed by degree of luminescence. Initially, validation between cell number and degree of luminescence was determined (confirmed *R*^2^ values ≥ 0.990, Figure 5A). Next, the proliferation of CRCT2-luc2 cells with or without NIH3T3 cells was analyzed by degree of luminescence. As a result, proliferation of CRTC2-luc2 cells was significantly upregulated in the co-cultured group, and tended to depend on the amount of NIH3T3 cells (Figure 5B). The effect of interaction between CRCT2-luc2 cells and NIH3T3, including secretion of growth factors, was suggested. Then, the therapeutic effect of MET-I in co-culture with different degrees of NIH3T3 was analyzed. Of interest, the effect was apparently reduced in the NIH3T3-enriched condition (ratio of initial cell numbers of CRCT2-luc2 and NIH3T3 is 1:2) compared with cultures without NIH3T3 or with the same number of NIH3T3 (Figure 5C). After preparation of HGF-knock down NIH3T3 cells (HGFKD#1, #2, Figure 5D), the effect of additional HGA-I was evaluated. No significant difference was apparent by the single use of MET-I under condition with NIH3T3 (ratio of CRTC2-luc2:NIH3T3 is 1:2); however, significant therapeutic effect was observed by MET-I in combination with HGFA-I (Figure 5E). In addition, similar therapeutic effect was also confirmed by the knock down of HGF in NIH3T3, suggesting the significance of the inhibition of fibroblast-induced HGF activation in proliferation of CRCT2-luc2 cell.

## 3. Discussion

Because liver metastasis has been reported to correlate with the worst OS in patients with CRPC, analysis to identify effective agents is necessary [5]. In PC, overexpression of MET has been reported in the metastatic site compared with the primary lesion, and expression is positively correlated with the progression of PC [23,24,25]. In addition, MET is highly expressed in androgen-independent CRPC cell lines, whereas it is downregulated in androgen-dependent PC cell lines [8,26]. Of interest, the degree of MET expression in the HSPC cell line is reported to continue increasing during the process to becoming an androgen-independent phenotype [26]. Therefore, MET is a key molecule in the progression of CRPC and may be a candidate for a target molecule in the treatment of androgen-independent CRPC. Indeed, a phase III study of atezolizumab plus cabozantinib, a potent multi-TKI including MET, for metastatic CRPC is currently underway [27]. Furthermore, serum concentration of HGFA and active HGF are also elevated in patients with CRPC compared with other stages [28,29].

Analysis of liver metastasis and HGF/MET signaling have been reported in gastrointestinal cancers. Overexpression of MET was observed in liver metastases of colorectal cancer and gastric cancer [30,31,32]. Expression of HGF was also evaluated in a study of gastric cancer [33]. HGF has been reported to be highly expressed in pericancerous tissues and the liver; however, not in metastatic cancer cells [33]. Since increased expression of MET and phosphorylation in cancer cells was confirmed, the data suggested that the metastatic cancer cells mainly bind with HGF, which is released into the pericancerous liver microenvironment in a paracrine manner [33]. In addition, the upregulation of liver HGF promoted metastasis to liver, the growth of metastases, increasing in size and weight of metastatic focus with increased phosphorylation of MET, whereas the downregulation of liver HGF suppressed liver metastasis [33]. As treatments, it has been reported that the downregulation of HGF/MET signaling by siRNA of MET or the expression of HGF-antagonist has achieved the suppression of liver metastasis in colorectal cancer [30,34].

In the current study, we initially established an androgen-independent mouse CRPC cell line, CRTC2, from the TRAMP-C2 cell line [20]. CRCT2 was used for the mouse model of liver metastasis by hemi-spleen injection, and apparent liver metastases were successfully formed and grew in a time-dependent manner, but no metastasis was observed in TRAMP-C2. Of interest, increased expression of HGF, MET, and two pro-HGF activating proteases (HGFA and hepsin) were observed in liver metastasis, which suggested that HGF/MET signaling may be a key system in liver metastasis of androgen-independent CRPC cells.

As specific agents, JNJ38877605 as MET-I and SRI31215 as HGFA-I were used for the inhibition of HGF/MET signaling. SRI31215 is a small molecule triplex inhibitor of HGFA, hepsin, and matriptase [35]. Combined therapy with both agents has been reported for MET-amplified NSCLC cells, and this therapy overcame resistance to MET inhibitors [35]. Our in vitro analysis revealed that HGFA-I clearly inhibited activation of pro-HGF and reduced phosphorylation of MET. In addition, combination with MET-I significantly reduced the growth of CRTC2 cells through the strongest inhibition of MET phosphorylation. In liver metastases, combined therapy with both MET-I and HGFA-I also showed the strongest and most significant inhibition of growth of liver metastases compared with control and single use of MET-I. Histological appearance also showed increased necrotic area and downregulation of MET phosphorylation in treatment groups. Weak phosphorylation of MET continued in single-use treatment; however, phosphorylation was completely downregulated by combined therapy. In the peripheral area of cancer nests in treatment groups, SMA positive stromal cells suggesting CAF presented around the viable cancer cells. Therefore, it is suggested that additional inhibition of pro-HGF activation is important for complete inhibition of MET because CAF-induced paracrine secretion of HGF may still be active. Basilico reported a significant effect with combined MET and HGF targeting therapy using a specific anti-MET antibody, which induced MET shedding (MET inhibition) with a decoy soluble extracellular domain of MET protein capable of binding HGF (HGF inhibition) [36]. Following analysis by orthotopic transplantation of human pancreatic carcinoma in mice engineered to express human HGF revealed significant reduction in metastatic spread, which also suggested the importance of combined therapy in HGF-rich condition. In addition, co-culture analysis with mouse fibroblast (NIH3T3), which expressed HGF, revealed that the effect of MET-I was reduced by co-culture with increased NIH3T3. However, the co-administration of HGFA-I (combined therapy) showed a significant reduction in cell growth, which suggested that blocking the HGF-paracrine loop between fibroblast and CRTC2-luc2 cells overcame resistance to MET-TKI.

## 4. Materials and Methods

### 4.1. Cell Culture and Reagents

Human prostate cancer LNCaP cells and mouse prostate cancer Tramp-C2 cells were purchased from the American Type Culture Collection (Manassas, VA, USA) and were maintained in RPMI-1640 media or Dulbecco’s Modified Eagle’s Medium (DMEM) containing 10% fetal bovine serum, 100 U/mL penicillin, and 100 µg/mL streptomycin (Gibco BRL, Grand Island, NY, USA) at 37 °C in a 5% CO_2_-humidified incubator. Cell numbers were counted using a Countess II (Invitrogen, Carlsbad, CA, USA).

### 4.2. Establishment of Mouse CRPC Cell Line (CRTC2)

According to the methodology in a previous report by Jeet et al. [20], we injected the Tramp-C2 cell line subcutaneously into 6-week-old female nude mice (Kyudo, Saga, Japan), and established the engrafted tumor as a CRPC cell line, CRTC2.

### 4.3. Real-Time Quantitative PCR (RT-qPCR)

Tissues were homogenized by a μT-1 (TAITEC, Nagoya, Japan) before extracting RNA. Total RNA of cells and tissue was extracted using PureLink RNA Mini Kit (Thermo Fisher Scientific, Waltham, MA, USA). cDNA was synthesized from total RNA using PrimeScript Reverse Transcriptase (Takara, Shiga, Japan) and random primers. Real-time RT-PCR analyses were performed with Thermal Cycler Dice Real-Time System II (Takara). Reaction mixture (25 μL) containing 2 μL cDNA template, 1 μL each of sense and anti-sense primers, and 1× SYBR Premix Ex Taq II (Takara) were amplified as follows: held at 95 °C for 30 s, then 40 cycles at 95 °C for 5 s, 60 °C for 3 s, followed by a final dissociation stage (95 °C for 15 s, 60 °C for 30 s, and 95 °C for 15 s). Glyceraldehyde-3-phosphate dehydrogenase (GAPDH) was used as an internal control. The results were evaluated using Thermal Cycler Dice Real Time System software program, version 5.11 (Takara Bio, Shiga, Japan), and the ΔΔCt algorithm was used to analyze relative changes in gene expression. The primers were as follows: mouse GAPDH forward, 5′-CATCACTGCCACCCAGAAGACTG-3′ and reverse, 5′-ATGCCAGTGAGCTTCCCGTTCAG-3′; mouse HGF forward, 5′-GTCCTGAAGGCTCAGACTTGGT-3′ and reverse, 5′-CCAGCCGTAAATACTGCAAGTGG-3′; mouse MET forward, 5′-GTTCTGCTTGGCAACGAGAGCT-3′ and reverse, 5′-GGAGAATGCACTGTATTGCGTCG-3′; mouse AR forward, 5′-CCTTGGATGGAGAACTACTCCG-3′ and reverse, 5′-TCCGTAGTGACAGCCAGAAGCT-3′; mouse Hepsin forward, 5′-ATACATCCAGCCAGTGTGTCTCC-3′ and reverse, 5′-CTCTTGGAGCACCATAGCCTGT-3′; mouse HGFA forward, 5′-TTCTGACCTGCTCTACCAGGAG-3′ and reverse, 5′-CGTTGTCCTTCACCACATAGCAC-3′; mouse; All experiments were performed in triplicate. Results of the PCR were presented with reference to the literature [37].

### 4.4. Microarray Analysis of Expression Molecules in CRTC2 and the Liver Metastasis

Total RNA was extracted from the sample using RNeasy Mini Kit (Qiagen, Hilden, Germany) according to the manufacturer’s instructions. The quality and quantity of the RNA were assessed using NanoDrop spectrophotometer (Thermo Fisher Scientific). Microarray analysis was outsourced to Cosmo Bio Co., Ltd. (Tokyo, Japan). The company performed cDNA synthesis, labeling, hybridization, washing, and scanning processes using Clariom S Array, mouse (Thermo Fisher Scientific) according to their proprietary protocols and equipment. The raw data provided by Cosmo Bio were normalized and analyzed using Transcriptome Analysis Console Software, Version 4.0.3 (Thermo Fisher Scientific). Genes with a fold-change of 2.0 or more were noted as differentially expressed.

### 4.5. Antibodies and Reagents

Anti-phospho-MET (#3077), and α-tubulin (#2125) antibodies were obtained from Cell Signaling Technology (Danvers, MA, USA). Anti-androgen receptor antibody was purchased from Merck (#06-680) (Rahway, NJ, USA), and anti-total MET antibody was obtained from Invitrogen (#PA5-85951) (Waltham, MA, USA). Anti-mouse HGF antibody was obtained from R&D Systems (Minneapolis, MN, USA) (#AF-2207). Recombinant mouse HGF propeptide protein (#7058-HG) and mouse HGF protein (#2207-HG) were purchased from R&D Systems. JNJ-38877605 (MET-I, tyrosine kinase inhibitor of MET) was obtained from Selleck Chemicals (#S1114) (Houston, TX, USA), and SRI31215 (HGF activator inhibitor: HGFA-I) was from MedChemExpress (Monmouth Junction, NJ, USA, #HY-114363A). Details of SRI31215, the hydrogen chloride salt of 3-(3-((1-benzylpiperidin-4-yl)methyl)-5-methyl-2-oxotetrahydropyrimidin-1(2H)-yl)benzimidamide, were described previously [38].

### 4.6. Protein Extraction and Immunoblot Analysis

Cells were washed twice with ice-cold PBS followed by incubation with the RIPA lysis buffer (Thermo Fisher Scientific) at 4 °C. The extracted protein was collected by centrifugation at 13,000 rpm for 15 min at 4 °C. Protein concentrations were determined by using the bicinchoninic acid method (Takara, Shiga, Japan). The reaction samples were mixed with sodium dodecyl sulfate–polyacrylamide gel electrophoresis (SDS-PAGE) sample buffer and heated for 15 min at 75 °C. SDS-PAGE was performed under reducing conditions using 4–12% gradient gels. After electrophoresis, the sample proteins were transferred electrophoretically to Immobilon membranes (Millipore; Billerica, MA, USA). After blocking the nonspecific binding site with PDVF Blocking Reagent (TOYOBO, Tokyo, Japan), the membranes were incubated with primary antibody in buffer containing 1% bovine serum albumin (BSA) at 4 °C overnight followed by four washes with the buffer and incubation with peroxidase-conjugated secondary antibody diluted in the buffer with 1% BSA for 1 h at room temperature. The labeled proteins were visualized with chemiluminescence reagent (PerkinElmer Life Sciences, Boston, MA, USA)

### 4.7. Proliferation Assay of Dual Inhibition of MET and HGF-Activation for CRTC2 Cells

In a 96-well plate, 1 × 10^4^ cells were seeded in 100 μL of medium and incubated for a specified period. Following this, 20 μL of CellTiter 96 Aqueous One Solution (Promega, Madison, WI, USA) was added to each well. The plate was then incubated for an additional 2 h at 37 °C, after which the absorbance of each well was measured at 490 nm. At 24 h post-seeding, the medium was replaced with FBS-free medium. The cells were then treated under conditions with or without MET-I and HGFA-I. Subsequently, pro-HGF was added at a concentration of 50 ng/mL. After a further incubation of 48 h, cell viability was assessed using 3-(4,5-dimethylthiazol-2-yl)-5-(3-carboxymethoxyphenyl)-2-(4-sulfophenyl)-2H-tetrazolium (MTS) assay. All experiments were performed in triplicate.

### 4.8. Transfection

Preparation of CRTC2-mCherry-luc2 (CRTC2-luc2) cells was performed. For stable gene transduction, we prepared lentivirus particles. To construct the CSII-CMV-MCS backbone plasmid (#RDB04377, RIKEN BRC, Saitama, Japan) for producing lentiviral particles carrying mCherry-luc2 reporter gene, we amplified mCherry-luc2 cDNA and inserted it into the NotI site of the CSII-CMV-MCS vector by using In-Fusion HD Cloning Kit (Takara Bio, Shiga, Japan). The CSII-CMV-mCherry-luc2 plasmid were co-transfected with the packaging plasmid psPAX2 (#12260, addgene, Watertown, MA, USA), the VSV-G-, and Rev-expressing plasmids (pCMV-VSV-G-RSV-Rev) (#RDB04393, RIKEN BRC) into Hek293T cells (CloneTech) by PEI MAX (Polysciences, Warrington, PA, USA). CRTC2 cells were cultured for 48 h in the medium with the lentivirus particles and 10 µg/µL polybrene (Sigma-Aldrich, St. Louis, MO, USA). The successfully transduced cells were isolated based on mCherry fluorescence and subsequently expanded. NIH3T3 cells were transfected with siRNA oligonucleotides targeting HGF (Thermo Fisher Scientific, Assay ID: s67510 and s67511) using Lipofectamine RNAiMAX (Thermo Fisher Scientific), and then HGF-knock down NIH3T3 cells, HGFKD#1 and #2 were prepared.

### 4.9. Co-Culture

CRTC2/luc2 cells (2.0 × 10^4^) were co-cultured with NIH3T3 in 100 μL of FBS-free medium. After 96 h, the medium was replaced with 50 μL of PBS, and 100 μL of D-luciferin (1 mM) was added. Luminescence was then measured at 30 s and the proliferation of CRCT2/luc2 was measured for average intensity. Validation between cell number and degree of luminescence was determined prior to proliferation assay.

### 4.10. Animal Experiments

All experiments involving laboratory animals were performed in accordance with the Guidelines for Experiments of Miyazaki University (Permit Number: 2023-522). Six-week-old male nude mice were purchased from Kyudo. Prior to the experiments, all mice underwent orchiectomy. According to the procedure by Simons BW [21], we established the mouse liver metastasis model by injecting the cell lines into spleen. Tramp-C2 or CRTC2 cells were cultured and then detached by trypsin EDTA at 80–90% confluence before resuspension in PBS to a final concentration of 2 × 10^6^ cells/100 µL. Mice were anesthetized with isoflurane inhalation, and Tramp-C2 or CRTC2 cells were injected into the spleen, followed by splenectomy.

For evaluation of the therapeutic effects of JNJ and SRI on liver metastasis, luciferase-expressing CRTC2-luc2 cells were injected into the spleen of castrated male nude mice.

The control group was given only vehicle, and the treatment groups received JNJ (30 mg/kg, oral administration, every other day) or JNJ with SRI (5 mg/kg, intravenous injection, every other day). For the bioimaging measurements, 100 mM of D-luciferin was injected intraperitoneally at a volume of 100 μL, the size range of the mouse liver was determined, and the luminescence count within this range was measured. Image acquisition was accomplished by using a Lumazone system equipped with an iKon L camera (Andor Technology Ltd., Belfast, UK) and a lens (YMV2595N, φ50 mm, f: 0.95, Yakumo Optical Corp., Tokyo, Japan) under the control of Andor Solis (Andor Technology Ltd., Belfast, UK). The following conditions were used for image lacquisition: exposure time = 1 or 5 s, binning = 4, readout rate = 50 kHz. The bioluminescence images were analyzed by MetaMorph (Molecular Devices LLC., Sunnyvale, CA, USA) and quantified photon counts per 1 or 5 s to draw the graphs. This luminescence count was measured 30–60 times, and the highest luminescence count was used as the average intensity. The temporal evaluation of the average intensity was calculated as a ratio to the value on the first day of drug administration.

### 4.11. Immunohistochemistry

Formalin-fixed paraffin-embedded sections were prepared according to the routine method. For immunohistochemistry, sections were processed for antigen retrieval (microwave in 10 mM citrate buffer, pH 6.0 for 10 min), followed by treatment with 3% H_2_O_2_ in methanol for 10 min and washed in tris-buffered saline (TBS) twice. After blocking in 3% bovine serum albumin and 5% goat serum in phosphatebuffered saline for 2 h at room temperature, the sections were incubated with primary antibodies overnight at 4 °C. Negative controls did not include the primary antibody. Sections were then washed in TBS and incubated with Envison labeled polymer reagent (DAKO, Santa Clara, CA, USA) for 30 min at room temperature. Sections were exposed to nickel, cobalt-3,3-diaminobenzidine (Immunopure Metal Enhanced DAB Substrate Kit; Piece, Rockford, IL, USA), and they were counterstained with hematoxylin.

### 4.12. Statistical Analysis

Differences between groups were compared using paired *t*-tests. Statistical tests were two-sided, and *p* values < 0.05 were considered statistically significant.

## 5. Conclusions

In the current study, we established androgen-independent CRPC cell line, CRTC2 and liver metastasis models in mice. We evaluated the therapeutic effect of HGF/MET-targeted agents. A significant effect was confirmed by combined treatment of MET inhibitor (MET-I) and inhibitor of HGF activation (HGFA-I) by in vitro and in vivo analysis. The possible role of CAF produced HGF paracrine system remained active by single-use treatment of MET-I suggesting the importance of combined treatment with both MET and HGF-targeting agent in the treatment of HGF enriched conditions, including liver metastasis.

## Data Availability

The data presented in this study are available on request from the corresponding author due to protection of participants’ privacy.

## Abbreviations

The following abbreviations are used in this manuscript:

CRPC	Castration-resistant prostate cancer
HGF	Hepatocyte growth factor
*HGFAC*	Hepatocyte growth factor activator coding gene
*HPN*	Hepsin coding gene
MET	HGF receptor
CAF	Cancer associated fibroblast
TKI	Tyrosine kinase inhibitor
NSCLC	Non-small cell lung cancer
CCA	Cholangiocarcinoma
SMA	Smooth muscle actin

## References

[1] James N.D., Tannock I., N’Dow J., Feng F., Gillessen S., Ali S.A., Trujillo B., Al-Lazikani B., Attard G., Bray F. (2024). The Lancet Commission on prostate cancer: Planning for the surge in cases. Lancet.

[2] Iwamoto H., Izumi K., Shimada T., Kano H., Kadomoto S., Makino T., Naito R., Yaegashi H., Shigehara K., Kadono Y. (2021). Androgen receptor signaling-targeted therapy and taxane chemotherapy induce visceral metastasis in castration-resistant prostate cancer. Prostate.

[3] Cetin K., Beebe-Dimmer J.L., Fryzek J.P., Markus R., Carducci M.A. (2010). Recent time trends in the epidemiology of stage IV prostate cancer in the United States: Analysis of data from the Surveillance, Epidemiology, and End Results Program. Urology.

[4] Fujimoto H., Nakanishi H., Miki T., Kubota Y., Takahashi S., Suzuki K., Kanayama H., Mikami K., Homma Y. (2011). Oncological outcomes of the prostate cancer patients registered in 2004: Report from the Cancer Registration Committee of the JUA. Int. J. Urol..

[5] Halabi S., Kelly W.K., Ma H., Zhou H., Solomon N.C., Fizazi K., Tangen C.M., Rosenthal M., Petrylak D.P., Hussain M. (2016). Meta-Analysis Evaluating the Impact of Site of Metastasis on Overall Survival in Men With Castration-Resistant Prostate Cancer. J. Clin. Oncol..

[6] Shirotake S., Umezawa Y., Okabe T., Kaneko G., Kanao K., Nishimoto K., Oyama M. (2020). A case of castration-resistant prostate cancer with liver metastases achieved a complete response by docetaxel chemotherapy. Transl. Androl. Urol..

[7] Bray A.W., Duan R., Malalur P., Drusbosky L.M., Gourdin T.S., Hill E.G., Lilly M.B. (2022). Elevated serum CEA is associated with liver metastasis and distinctive circulating tumor DNA alterations in patients with castration-resistant prostate cancer. Prostate.

[8] Mukai S., Yamasaki K., Fujii M., Nagai T., Terada N., Kataoka H., Kamoto T. (2020). Dysregulation of Type II Transmembrane Serine Proteases and Ligand-Dependent Activation of MET in Urological Cancers. Int. J. Mol. Sci..

[9] Kawaguchi M., Kataoka H. (2014). Mechanisms of hepatocyte growth factor activation in cancer tissues. Cancers.

[10] Kataoka H., Kawaguchi M., Fukushima T., Shimomura T. (2018). Hepatocyte growth factor activator inhibitors (HAI-1 and HAI-2): Emerging key players in epithelial integrity and cancer. Pathol. Int..

[11] Owusu B.Y., Thomas S., Venukadasula P., Han Z., Janetka J.W., Galemmo Jr R.A., Klampfer L. (2017). Targeting the tumor-promoting microenvironment in MET-amplified NSCLC cells with a novel inhibitor of pro-HGF activation. Oncotarget.

[12] Moosavi F., Giovannetti E., Peters G.J., Firuzi O. (2021). Combination of HGF/MET-targeting agents and other therapeutic strategies in cancer. Crit. Rev. Oncol. Hematol..

[13] Suda K., Mizuuchi H., Maehara Y., Mitsudomi T. (2012). Acquired resistance mechanisms to tyrosine kinase inhibitors in lung cancer with activating epidermal growth factor receptor mutation—Diversity, ductility, and destiny. Cancer Metastasis Rev..

[14] Owusu B.Y., Galemmo R., Janetka J., Klampfer L. (2017). Hepatocyte Growth Factor, a Key Tumor-Promoting Factor in the Tumor Microenvironment. Cancers.

[15] Pennacchietti S., Cazzanti M., Bertotti A., Rideout W.M., Han M., Gyuris J., Perera T., Comoglio P.M., Trusolino L., Michieli P. (2014). Microenvironment-derived HGF overcomes genetically determined sensitivity to anti-MET drugs. Cancer Res..

[16] Affo S., Yu L.X., Schwabe R.F. (2017). The Role of Cancer-Associated Fibroblasts and Fibrosis in Liver Cancer. Annu. Rev. Pathol. Mech. Dis..

[17] Ilyas S.I., Affo S., Goyal L., Lamarca A., Sapisochi G., Yang J.D., Gores G.J. (2023). Cholangiocarcinoma—Novel biological insights and therapeutic strategies. Nat. Rev. Clin. Oncol..

[18] Affo S., Filliol A., Gores G.J., Schwabe R.F. (2023). Fibroblasts in liver cancer: Functions and therapeutic translation. Lancet Gastroenterol. Hepatol..

[19] Affo S., Nair A., Brundu F., Ravichandra A., Bhattacharjee S., Matsuda M., Chin L.K., Filliol A., Wen W., Song X. (2021). Promotion of cholangiocarcinoma growth by diverse cancer-associated fibroblast subpopulations. Cancer Cell.

[20] Jeet V., Ow K., Doherty E., Curley B., Russell P.J., Khatri A. (2008). Broadening of transgenic adenocarcinoma of the mouse prostate (TRAMP) model to represent late stage androgen depletion independent cancer. Prostate.

[21] Simons B.W., Dalrymple S., Rosen M., Zheng L., Brennen W.N. (2020). A hemi-spleen injection model of liver metastasis for prostate cancer. Prostate.

[22] Biffi G., Tuveson D.A. (2021). Diversity and biology of cancerassociated fibroblasts. Physiol. Rev..

[23] Janetka W.J., Benson M.R. (2018). Extracellular Targeting of Cell Signaling in Cancer.

[24] Knudsen B.S., Gmyrek G.A., Inra J., Scherr D.S., Vaughan E.D., Nanus D.M., Kattan M.W., Gerald W.L., Vande Woude G.F. (2002). High expression of the Met receptor in prostate cancer metastasis to bone. Urology.

[25] Verhoef E.I., Kolijn K., De Herdt M.J., van der Steen B., Hoogland A.M., Sleddens H.F., Looijenga L.H., van Leenders G.J. (2016). MET expression during prostate cancer progression. Oncotarget.

[26] Nakashiro K., Hara S., Shinohara Y., Oyasu M., Kawamata H., Shintani S., Hamakawa H., Oyasu R. (2004). Phenotypic switch from paracrine to autocrine role of hepatocyte growth factor in an androgen-independent human prostatic carcinoma cell line, CWR22R. Am. J. Pathol..

[27] Agarwal N., Azad A., Carles J., Chowdhury S., McGregor B., Merseburger A.S., Oudard S., Saad F., Soares A., Benzaghou F. (2022). A phase III, randomized, open-label study (CONTACT-02) of cabozantinib plus atezolizumab versus second novel hormone therapy in patients with metastatic castration-resistant prostate cancer. Future Oncol..

[28] Sugie S., Mukai S., Yamasaki K., Kamibeppu T., Tsukino H., Kamoto T. (2016). Plasma macrophage-stimulating protein and hepatocyte growth factor levels are associated with prostate cancer progression. Hum. Cell.

[29] Nagakawa O., Yamagishi T., Fujiuchi Y., Junicho A., Akashi T., Nagaike K., Fuse H. (2005). Serum hepatocyte growth factor activator (HGFA) in benign prostatic hyperplasia and prostate cancer. Eur. Urol..

[30] Yao J.F., Li X.J., Yan L.K., He S., Zheng J.B., Wang X.R., Zhou P.H., Zhang L., Wei G.B., Sun X.J. (2019). Role of HGF/c-Met in the treatment of colorectal cancer with liver metastasis. J. Biochem. Mol. Toxicol..

[31] Amemiya H., Kono K., Itakura J., Tang R.F., Takahashi A., An F.Q., Kamei S., Iizuka H., Fujii H., Matsumoto Y. (2002). c-Met expression in gastric cancer with liver metastasis. Oncology.

[32] Elliott V.A., Rychahou P., Zaytseva Y.Y., Evers B.M. (2014). Activation of c-Met and upregulation of CD44 expression are associated with the metastatic phenotype in the colorectal cancer liver metastasis model. PLoS ONE.

[33] Zhang H., Deng T., Liu R., Bai M., Zhou L., Wang X., Li S., Wang X., Yang H., Li J. (2017). Exosome-delivered EGFR regulates liver microenvironment to promote gastric cancer liver metastasis. Nat. Commun..

[34] Wen J., Matsumoto K., Taniura N., Tomioka D., Nakamura T. (2004). Hepatic gene expression of NK4, an HGF-antagonist/angiogenesis inhibitor, suppresses liver metastasis and invasive growth of colon cancer in mice. Cancer Gene Ther..

[35] Owusu B.Y., Bansal N., Venukadasula P.K., Ross L.J., Messick T.E., Goel S., Galemmo R.A., Klampfer L. (2016). Inhibition of pro-HGF activation by SRI31215, a novel approach to block oncogenic HGF/MET signaling. Oncotarget.

[36] Basilico C., Modica C., Maione F., Vigna E., Comoglio P.M. (2018). Targeting the MET oncogene by concomitant inhibition of receptor and ligand via an antibody-“decoy” strategy. Int. J. Cancer.

[37] So S., Park Y., Kang S.S., Han J., Sunwoo J.H., Lee W., Kim J., Ye E.A., Kim J.Y., Tchah H. (2022). Therapeutic Potency of Induced Pluripotent Stem-Cell-Derived Corneal Endothelial-like Cells for Corneal Endothelial Dysfunction. Int. J. Mol. Sci..

[38] Venukadasula P.K.M.O.B., Bansal N., Ross L.J., Hobrath J.V., Bao D., Truss J.W., Stackhouse M., Messick T.E., Klampfer L., Galemmo R.A. (2016). Design and Synthesis of Nonpeptide Inhibitors of Hepatocyte Growth Factor Activation. ACS Med. Chem. Lett..

